# Spironolactone and Cardiac Arrhythmias in Hemodialysis Patients: Helpful or Harmful?

**DOI:** 10.34067/KID.0000000000000096

**Published:** 2023-04-27

**Authors:** Patrick H. Pun

**Affiliations:** 1 Division of Nephrology, Department of Medicine, Duke University School of Medicine, Durham, North Carolina; 2 Duke Clinical Research Institute, Durham North Carolina

**Keywords:** cardiovascular disease, cardiovascular events, chronic dialysis

Hemodialysis patients are subject to extraordinarily high risks of fatal and nonfatal cardiac arrhythmias; the annual risk of sudden death exceeds the general population risk by nearly 20-fold.^1^ While improvements in cardiovascular disease management in the ESKD population over the past two decades has resulted in measurable improvements in the overall risk of cardiovascular death, similar gains have not been achieved in sudden death, which now accounts for an increasing proportion of all deaths among hemodialysis patients.^2^ Thus, there is an urgent need for interventions that will reduce the risk of sudden death in the dialysis-dependent ESKD population.

Besides aldosterone's well-characterized effects on sodium and potassium handling in the kidney, it also mediates fibrotic and hypertrophic effects on the heart and stimulates proinflammatory cytokines.^3^ Aldosterone receptor antagonists, such as spironolactone, have been proven to reduce sudden death among patients with heart failure and reduced ejection fraction and are, therefore, attractive potential treatments to decrease arrhythmogenesis in patients with ESKD who are known to have markedly elevated aldosterone levels, excess myocardial fibrosis, and pathologic left ventricular remodeling.^4,5^ However, owing to enhanced fecal potassium losses in ESKD and the presence of aldosterone receptors in the colonic epithelium, aldosterone antagonists can promote hyperkalemia even among anephric participants.^6^ Dialysis patients are already subject to wide swings in serum potassium levels, which has been linked to the risk of sudden death in patients with ESKD, and aldosterone antagonists could further disrupt potassium homeostasis.^7^ While multiple small randomized studies of aldosterone antagonists in dialysis-dependent ESKD have provided some reassurance that the risk of severe hyperkalemia (*i.e.*, >6.5 mEq/L) is not increased,^3^ it is notable that potassium monitoring in these studies was generally infrequent once a steady dose was achieved (*i.e.*, once a month), leading to the possibility of undetected hyperkalemia. Furthermore, data on risks of significant cardiac arrhythmia and sudden death were absent or limited. In sum, the net benefit of aldosterone antagonists to decrease the risk of arrhythmias in hemodialysis patients is unclear.

In this issue of *Kidney360*, Dr. McCausland and colleagues provide data on the effects of spironolactone on arrhythmias for patients on hemodialysis by conducting a secondary analysis of the Safety and Cardiovascular efficacy of Spironolactone in dialysis-dependent ESKD (SPIN-D) randomized control trial.^8^ The primary objective of the double-blinded, placebo-controlled study was to evaluate the safety and tolerability of a range of spironolactone dosages among patients receiving maintenance hemodialysis and secondarily to provide preliminary estimates of efficacy on cardiac remodeling parameters. The primary study did not detect a statistically significant increase in the overall incidence of hyperkalemia between the placebo and intervention arms, although a numerically higher number of hyperkalemic events occurred in the 50 mg per day spironolactone arm compared with placebo. In addition, there was no detectable difference in the measured cardiac outcomes between the intervention and placebo arms at the end of the 36-week follow-up period.

The present study examines a subset of SPIN-D participants (57 of 129 total participants) who underwent 7 days of continuous heart rate and rhythm monitoring using a wearable patch at baseline, 6 weeks, and study end. The authors classified the arrhythmias detected and compared event rates according to treatment assignments at each time point. Importantly, most of the participants had arrhythmia data only available at study end as compared with the earlier periods, limiting the ability to control for the presence of baseline arrhythmias and also evaluate the consistency of findings throughout the study. In summary, there were no major differences in baseline characteristics between the intervention groups and placebo. Consistent with other monitoring studies, supraventricular arrhythmias were the most common (atrial fibrillation/flutter and atrial tachycardia), followed by bradycardia or conduction blocks; ventricular arrhythmias were rare (only three detected in the entire study). At baseline, there were more participants with events and higher arrhythmia event rates in the placebo arm, whereas there were more participants with events and a higher crude event rate in the spironolactone arms at 6 weeks and study end, driven primarily by higher rates of bradycardia and conduction block. At the end of follow-up, the minimally adjusted rate ratio for risk of bradycardia or conduction block in the spironolactone arm was twice as high as in the combined spironolactone groups compared with placebo; however, due to the small sample size, the 95% confidence interval (CI) was wide and included unity (2.04; 95% CI, 0.83 to 5.05). Additional mechanistic analyses were attempted to examine the association between exposures of interest (recent hyperkalemia and serum potassium values) with risk of arrhythmias. Recent hyperkalemia was associated with a rate ratio of 2.07 for bradycardia/conduction block, but the result was not statistically significant (95% CI, 0.52 to 8.25). Once again, it is important to note that serum potassium measurements were only obtained monthly at study end, so the rarity of available serum potassium data near the time of arrhythmia detection further hampers interpretation because of the lack of statistical power.

Overall, given the estimates of increased risk for bradycardia/conduction block associated with spironolactone and hyperkalemia, this study at least provides a rationale for concern; however, the results were clearly underpowered and nonsignificant, making it just as likely that the risk estimates were obtained by chance. Thus, it is difficult to know what to conclude from the study findings, except that the benefit/risk of aldosterone antagonists among hemodialysis patients is still in question. Being mindful of our history of being too quick to jump on or off bandwagons that are propelled by results in nondialysis populations and small clinical trials, it is too soon to conclude that spironolactone will achieve net benefits or harms. Fortunately, two large outcomes-powered trials (ALCHEMIST: ClinicalTrials.gov identifier NCT01848639; ACHIEVE: ClinicalTrials.gov identifier NCT03020303) are concluding soon, so we may have better answers shortly. Until then, although concerns raised by this study may not be borne out by definitive data, it may be prudent to err on the side of safety by being more attentive to potassium levels and the potential for bradycardia whenever spironolactone is prescribed, especially at higher doses.
